# Quantitative Proteomic analysis on Activated Hepatic Stellate Cells reversion Reveal STAT1 as a key regulator between Liver Fibrosis and recovery

**DOI:** 10.1038/srep44910

**Published:** 2017-03-21

**Authors:** Hongyu Zhang, Fangyan Chen, Xu Fan, Cong Lin, Yunwei Hao, Handong Wei, Weiran Lin, Ying Jiang, Fuchu He

**Affiliations:** 1 State Key Laboratory of Proteomics, National Center for Protein Sciences Beijing, Beijing Proteome Research Center, Beijing Institute of Radiation Medicine, Beijing 102206, China; 2 School of Life Sciences, Tsinghua University, Beijing, 100084, P. R. China; 3 State Key Laboratory of Space Medicine Fundamentals and Application, China Astronaut Research and Training Center, Beijing, 100094, P. R. China

## Abstract

Understanding the changes of activated HSCs reversion is an essential step toward clarifying the potential roles of HSCs in the treatment of liver fibrosis. In this study, we chose adipocyte differentiation mixture to induce LX-2 cells for 2 days *in vitro* as reversion phase, comparing with normal cultured LX-2 cells as activation phase. Mass spectrometric-based SILAC technology was adopted to study differentially expressed proteome of LX-2 cells between reversion and activation. Compared with activated HSCs, 273 proteins showed significant differences in reverted HSCs. The main pathway of up-regulated proteins associated with reversion of HSCs mainly related to oxidation-reduction and lipid metabolism, while the top pathway of down-regulated proteins was found in regulated cytoskeleton formation. Changes in the expression levels of selected proteins were verified by Western blotting analysis, especially STAT1, FLNA, LASP1, and NAMPT proteins. The distinct roles of STAT1 were further analyzed between activated and reverted of HSCs, it was found that STAT1 could affect cell proliferation of HSCs and could be viewed as a key regulator in the reversion of HSCs. Thus, the proteomic analysis could accelerate our understanding of the mechanisms of HSC reversion on cessation of fibrogenic stimuli and provide new targets for antifibrotic liver therapy.

The hepatic stellate cell, first described by Kupffer in 1876, is vital to hepatocellular function and the liver’s response to injury^1^,^2^. In normal liver, HSCs play a key role in the storage and transport of retinoids (vitamin A compounds). Upon liver injury (e.g., hepatitis B virus, hepatitis C virus, biliary obstruction, or alcohol), HSC are activated with the conversion of a resting vitamin A-rich lipid cell to one that is proliferating, fibrogenic, and contractile^3^. When the etiological source (e.g., hepatitis virus) is removed, liver fibrosis could regress associated with decrease of cytokine and ECM production, increase of collagenase activity, and the disappearance of activated HSC^1^,^4^. Although the mechanism of HSC activation has been comprehensively studied, insights into the fate of HSCs during recovery of liver fibrosis are few reported and paid increasing attention^5^. So far, there is no ideal model to study the reversion process of HSCs *in vitro*. However, She H *et al*.^6^ tested the effects of the adipocyte differentiation mixture (isobutylmethylxanthine, dexamethazone, and insulin (MDI)) on activated HSC and suggested that MDI could convert activated HSCs to fat storage state even quiescent phenotype. The human hepatic cell line LX-2 was generated by XU L *et al*.^7^. It was spontaneous immortalization with the Simian Vacuolating Virus 40 (SV40) transforming antigen and propagation in low serum and have extensively validated their similarities to human culture activated stellate cells^8^. Such stable, immortal cell lines could as tool to study mechanisms of hepatic fibrogenesis and test antifibrotic compounds. Therefore, availability of MDI-induced model of changes in HSCs *in vitro* could help to understand the trans-differentiation process of HSCs reversion during liver fibrosis.

In this study, we chose MDI mixture to induce culture LX-2 cell line for 2 days *in vitro* as reversion phase, comparing with normal cultured LX-2 *in vitro* as activation phase. To this end, stable isotope labeling with amino acids in cell culture (SILAC) was used in LX-2 cells as a straightforward and accurate approach and widely used for the relative quantification of cellular proteins, based on the metabolic incorporation of nonradioactive stable “heavy” and “light” isotopes^9^. Heavy and light peptides were distinguished by MS analysis and protein abundances were determined from relative MS signal intensities^10^. Data identification and quantification was high accurately performed using MaxQuant (MQ) software with the specifically developed algorithms in combination with the Mascot search engine^11^,^12^.

For our experiments, SILAC-labeled LX-2 cells in culture were first used and a differentially expressed proteome analysis was performed on cellular proteins between MDI-induced reverted hepatic stellate cells and activated hepatic stellate cells. Through Maxquant software analysis, 1347 proteins of 2293 total cell proteins were quantitated and there were 212 up-regulated proteins and 61 down-regulated proteins. These identified proteins should be useful for the characterization of hepatic stellate cells in different cultures and help us to explain whether activated HSC can reverse to quiescent-like HSCs, whether exists key proteins or pathways during HSC reversion, and whether they affect the hepatic response to chronic injury. Furthermore, the findings presented here would provide a solid foundation for new and more functionally oriented projects, which could contribute to the development of effective therapies against liver fibrosis.

## Results

### Confirmation of quiescent characteristics of MDI-induced reverted model of LX-2 cells

According to Tsukamoto’s method, the treatment of activated HSC with the adipocyte differentiation mixture (isobutylmethylxanthine, dexamethasone, and insulin MDI) could induce the phenotypic revert to quiescent HSCs. MDI or MDI with 10% FBS medium were chosen to culture LX-2 cells to confirm quiescent characteristics of HSC reversion model^6^. After exposed in MDI for 1 day or in MDI with 10% FBS for 2 days, LX-2 cells accumulated similarly more intracellular lipid than normal culture in accordance with the observation of morphological change (Supplemental Figure 1A) and the assessment of Lipid accumulation by Oil Red O staining (Supplemental Figure 1B) in LX-2 cells. Whereafter, Cell counting kit-8 was conducted to assess cell proliferation and discovered LX-2 cells proliferation potential were decreased both in MDI for 1 day and in MDI with 10% FBS for 2 days; Similarly, the PI (proliferative index, PI = S + G2/M)of LX-2 cells cultured both in MDI for 1 day and in MDI with 10%FBS for 2 days was lower than that of control groups (*p* < 0.01, *p* < 0.05). However the G0/G1 phase ratio of LX-2 cells cultured both in MDI for 1 day and in MDI with 10% FBS for 2 day was higher than that of control groups (*p* < 0.01, *p* < 0.05) (Supplemental Figure 1C).

In general, α-SMA, VIM and DES (Desmin) are known as markers of activated HSCs. However, there is no DES protein in human body. Therefore α-SMA and VIM expression were decreased in LX-2 cells cultured both in MDI for 1 day and in MDI with 10% FBS for 2 day using Western blotting (Supplemental Figure 1D). Thus, in the present study, LX-2 cells cultured both in MDI for 1 day and in MDI with 10% FBS for 2 days showed quiescent characters of HSCs and referred to revert their myofibroblast phenotype to that of quiescent HSCs. Because the results of LX-2 cells cultured in MDI with 10% FBS for 2 days were more satisfied than that in MDI for 1 day, LX-2 cells cultured in MDI with 10% FBS for 2 day were used for follow-up research.

### Differentially expressed proteins between reverted and activated HSCs revealed by SILAC analysis

**Figure 1:**
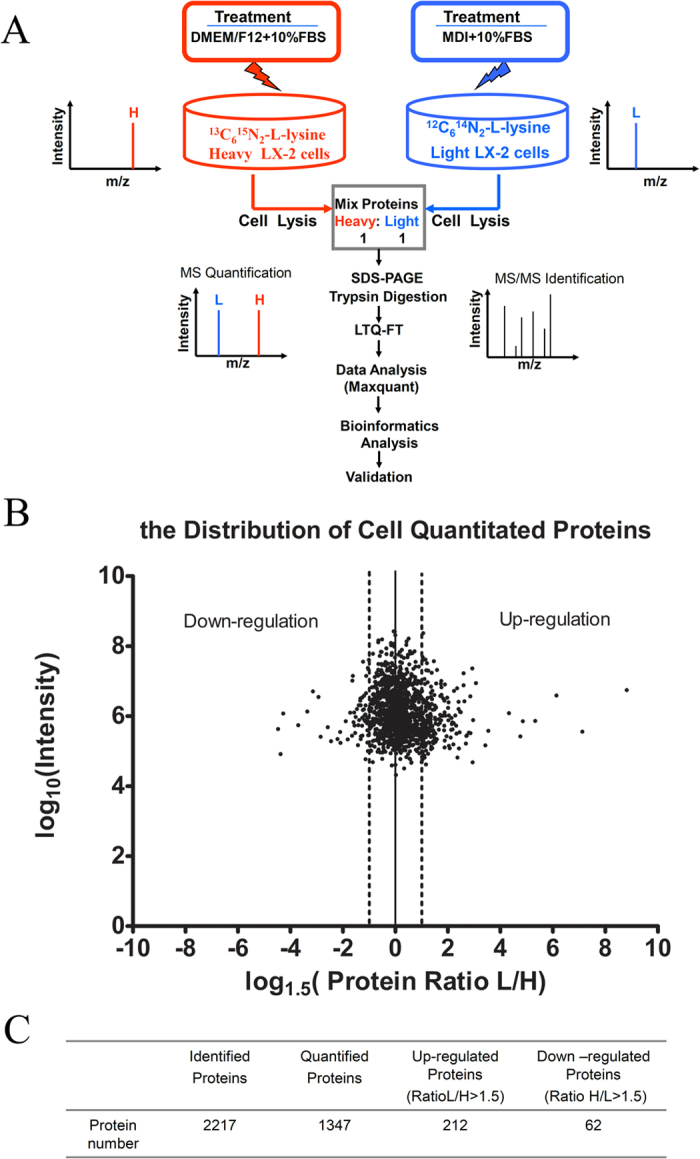
Schematic overview of the study design and summarizing the results. (**A**) Schematic overview of the SILAC method combined with LTQ-FT analysis used in this study. (**B**) The summary of the number of proteins identified and quantified in SILAC experiment and proteins with ratio (L/H) or ratio (H/L) greater than 1.5 were considered as significantly perturbed by MDI. (**C**) Distribution of protein expression ratios as determined by SILAC (n = 1,347 proteins, mean SILAC ratio = 0.97). Note: SILAC data was analysed by Analyst Maxquant (version 1.1.1.36). L: MDI group, H: Con group.

In the present study, SILAC was applied to gain insight into the molecular mechanisms of MDI-induced reverted HSCs at the protein level and evaluate the differential proteome between activated and reverted HSCs for the first time. A schematic overview of the study design is presented in Fig. 1A. To characterize differential proteins, MDI-induced reverted and activated LX-2 cells were labeled with ^12^C_6_^14^N_2_ and ^13^C_6_^15^N_2_ lysine, respectively. After separating the protein mixture by SDS-PAGE, the gel line was excised into 52 sections in order to identify more proteins with low abundance. Subsequently, the samples were digested with trypsin and analyzed by LC/MS. Using MaxQuant 1.1.1.36 software, 2217 proteins were identified (FDR < 1%) with no protein found in reverse database in according to the strict criteria described above, underlying that the data were reliable. From these, 1347 proteins of relative quantification data was obtained with at least two identified lysine-containing peptides. In Fig. 1B, Ratios of most quantitated proteins distributed approximately 1.0 (log_1.5_ ratio = 0), indicating that the cell proteins of MDI-induced reverted and activated LX-2 cells were mixed equally. Of these, 274 proteins showed a 1.5 fold or higher change, including 212 up-regulated and 62 down-regulated proteins in MDI-induced reverted model compared to activated LX-2 cells (Fig. 1C, Supplemental Tables 1 and 2).

### Bioinformatic analysis of differentially expressed proteins

**Figure 2:**
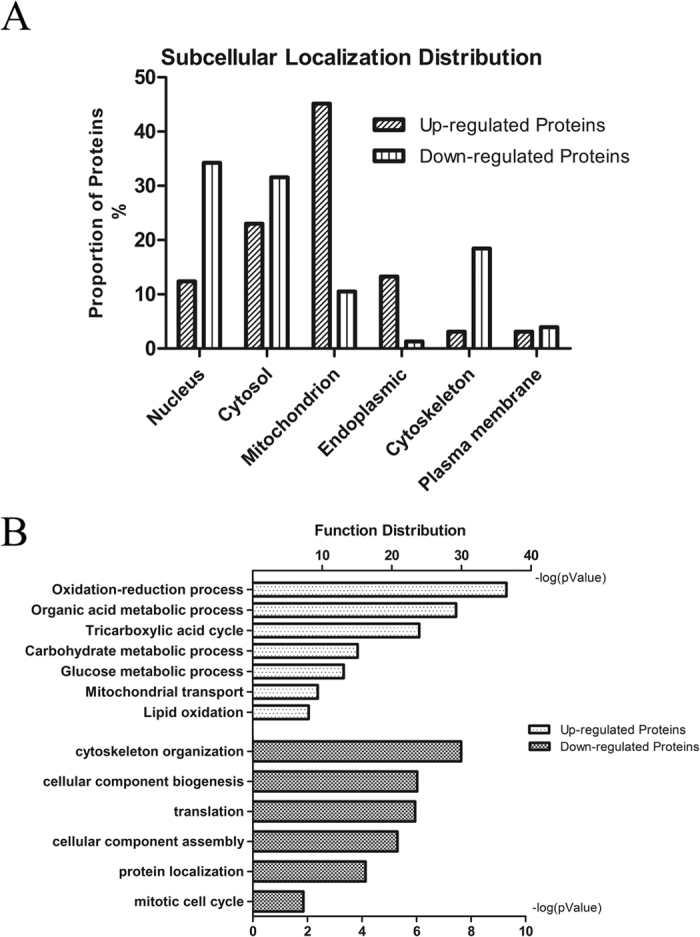
Subcellular and biological function of differential expressed proteins during HSCs revertion. (**A**) Subcellular localization analysis of altered proteins during LX-2 cells reversion. (**B**) Biological function analysis of differential proteins during LX-2 cells reversion. The top biological functions of the up-regulated and down-regulated proteins were determined by MetaCore software analysis from Thomson Reuters (http://portal.genego.com/), as shown. The x axis shows the negative log of *p* value.

The 274 altered proteins were annotated in cellular component and biological process analysis by MetaCore software (https://portal.genego.com/). It revealed that most of MDI-induced up-regulated proteins were localized in mitochondrion and cytosol while most of down-regulated proteins mainly concentrated in nucleus, cytosol and cytoskeleton, indicating that differentially expressed proteins are significant at the subcellular level (Fig. 2A). The biological function analysis on these altered proteins showed that MDI-induced reverted LX-2 cells experienced significant up-regulation in oxidation-reduction process, organic acid metabolic process, tricarboxylic acid cycle, carbohydrate metabolic process, glucose metabolic process, mitochondrial transport and lipid oxidation process, indicative of adipogenic transdifferentiation of reverted HSCs. However, proteins associated with cytoskeleton organization, cellular component biogenesis, translation, cellular component assembly, protein localization, and mitotic cell cycle were remarkably down-regulated in accord with myfibroblastic HSCs’ biological characteristics, as shown in Fig. 2B. Therefore, it was firstly discovered that both lipid metabolism and skeleton organization suppressed each other in HSCs, suggesting that it is necessary to study further the mechanism of their interaction.

### Validation of the Proteomic Results in LX-2 cell line and mice tissue

**Figure 3:**
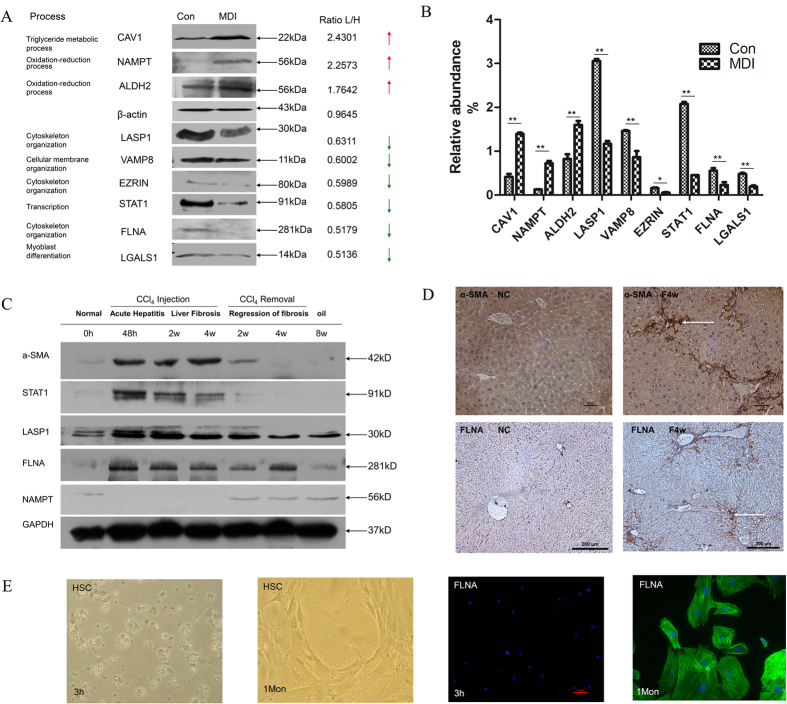
Western blotting validation of selected proteins and validation of key molecules by carbon tetrachloride (CCl_4_)-induced liver injury mouse model. (**A**) Differential expression of 3 up-regulated proteins (CAV1, NAMPT, ALDH2) and 6 down-regulated proteins (STAT1, EZRIN, LGALS1, LASP1, VAMP8 and FLNA) was validated by Western blotting. The relative ratios of proteins by SILAC analysis are shown at right. (**B**) Relative expression levels were normalized to β-actin. Representative blots from three independent experiments are shown. *p < 0.05; **p < 0.01 compare with Con; Con and MDI represent activated and MDI reverted LX-2 cells; L: MDI group, H: Con group. (**C**) Western blotting analysis of liver tissue for the levels of α-SMA, STAT1, LASP1, Filamin-A (FLNA), Nampt proteins during normal, acute hepatitis, liver fibrosis, recovery and oil-treated normal phase in carbon tetrachloride (CCl_4_)-induced mouse model, GAPDH was used as loading control. (**D**) Immunohistochemistry of α-SMA and FLNA in normal and fibrosis stage in CCl_4_-induced mouse model. Each image is a representative of six animals in each group. (**E**) The expression of FLNA in active mouse hepatic stellate cells (I) bright field, Bunches of cytoplasmic lipid droplets in primary quiescent HSCs cultured for 3 hours (original magnification 200×). (II) bright field, Depletion of cytoplasmic lipid droplets in activated HSCs cultured for 1 month (original magnification 200×). (III) Immunofluorescent staining of FLNA in quiescent HSCs cultured for 3 hours (FITC, green) (original magnification 400×). Quiescent HSCs did not express FLNA, (IV) Immunofluorescent staining of FLNA in activated HSCs cultured for 1 month (FITC, green) (original magnification 400×). Activated HSCs expressed FLNA.

In order to validate the results from SILAC-based proteomic analysis, Western blotting analysis was carried out for nine representative proteins in LX-2 cell line, focusing on those involved in the lipid metabolism and skeleton organization process of HSCs. Compared with activated HSCs, the three proteins involved in triglyceride metabolic process (CAV1), oxidation-reduction process (NAMPT and ALDH2) showed a remarkable up-regulation; whereas the expression of six proteins involved in cytoskeleton organization (FLANA, EZRIN, LASP1), cellular membrane organization (VAMP8), myoblast differentiation (LGALS1), transcription (STAT1) displayed remarkable down-regulation in MDI-induced reverted LX-2 cells. The Western blotting results for these molecules showed the same differential trend to the SILAC data (Fig. 3A).

For further validation, the C57BL6 mouse model of carbon tetrachloride (CCl_4_) -induced liver fibrosis was established as described^13^, which was verified with sirius red staining in formalin/paraffin sections (Supplemental Figure 2). Differential expression of selected proteins was further evaluated by Western blotting in this *in vivo* model, and found the proteins (α-SMA, STAT1, LASP1 and FLNA) increased in acute hepatitis (48 hours) and fibrosis phase (CCl_4_ injection, 2 weeks and 4 weeks) but decreased in recovery phase (CCl_4_ removal, 2 weeks and 4 weeks) comparing with normal mice liver tissue (olive given) and recovery phase (CCl_4_ removal, 2 weeks and 4 weeks). Whereas NAMPT protein showed reverse expression pattern as displayed in Fig. 3C. The expression of cytoskeletal protein (Filamin-A) was further found increased in CCl_4_-treated mice liver tissue of fibrosis phase (4 weeks) by IHC or *in vitro* cultured activated HSCs (1 month) by immuno-fluorescent staining comparing with normal mice liver tissue (olive given) or quiescent HSCs (3 h) respectively (Fig. 3D,E). These results further clarify the status and role of these proteins in liver fibrosis reversion.

### The Biological Significance of STAT1 in reverted and activated HSCs

**Figure 4:**
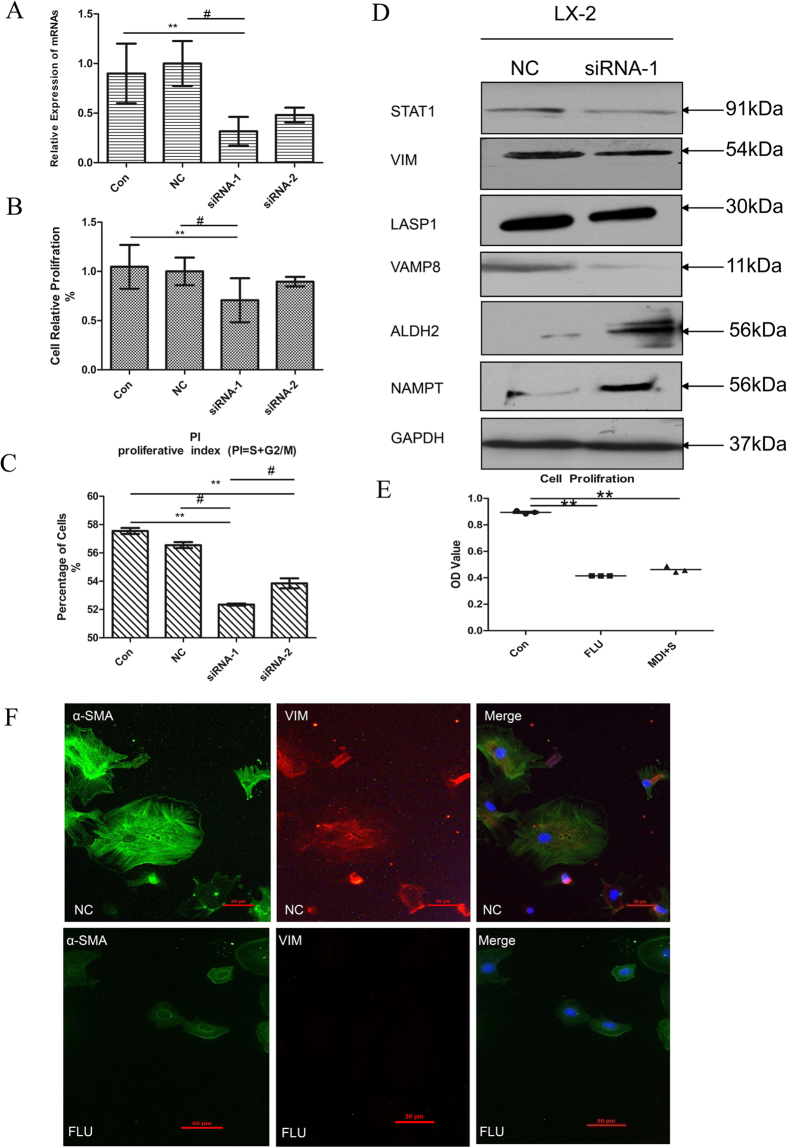
Involvement of STAT1 in HSC proliferation, regulating skeleton proteins and metabolism-related proteins. (**A**) Knockdown efficiency of siRNAs. LX-2 cells were transfected with STAT1 specific siRNA or with NC siRNA, after 48 h, their mRNA and protein levels were determined by qPCR, GAPDH is used as a loading control, n = 3. (**B** and **C**) Knockdown of STAT1 suppresses the proliferation of LX-2 cells. (**B**) cell proliferation. The mean OD value of cell proliferation was lower in siRNA-1 group (*p* < 0.01, *p* < 0.05) than that in the control group and NC group respectively. (**C**) the PI (proliferative index, PI = S + G2/M) of siRNA-1 group (*p* < 0.01, *p* < 0.05) was lower than that of control groups and NC group respectively. ^#^*p* < 0.05 compare with NC; **p < 0.01 compare with Con, n = 3. (**D**) Knockdown of STAT1 regulating LX-2 cells skeleton proteins and metabolism-related proteins expression by Western blotting. n = 3. (**E** and **F**) Involvement of Fludarabine (FLU) in HSC proliferation, regulating skeleton proteins. (**E**) The proliferation of LX-2 cells were significantly inhibited after treated with FLU (50 μM) for 48 h as MDI. **p < 0.01 compared with Con, n = 3. (**F**) FLU (50 μM) reduces the expression of α-SMA and Vimentin protein in primary mouse activated HSCs, n = 3. Note: Con, the untransfected normal cells; NC, Normal control; FLU, Fludarabine.

In the present study, the expression of STAT1 protein was reported to display decreased in reverted HSCs for the first time. Until now, however, there were very few reports about STAT1 function in activated HSCs. So it was evaluated that the biological significance of STAT1 proteins in activated HSCs in siRNA transfection experiments (Fig. 4A). Cell counting was conducted to assess cell proliferation and the data showed that knockdown of STAT1 significantly suppressed the proliferation of activated HSCs as well as the cell cycle of activated HSCs is limited (Fig. 4B and C). Furthermore, knocking down STAT1 could suppress the expression of Vimentin, LASP1 and VAMP8 proteins, but promote the expression of ALDH2 and NAMPT proteins in LX-2 cells (Fig. 4D).

STAT1 specific inhibitor – fludarabine (FLU) was also chose to analyze the function of STAT1. Our data showed that the proliferation of LX-2 cells were also significantly inhibited (Fig. 4E) and the expression of α-SMA and VIM proteins were limited in primary activated HSCs (Fig. 4F) after treated with FLU for 48 h. It indicated that FLU mediated inhibition of STAT1 significantly suppressed the proliferation and fibrogenic gene expression of HSCs. Thus, in the present study, it was illustrated that STAT1 has the distinct roles in proliferation and differentiation of HSCs.

## Discussion

### The proteomic view of differentially expressed proteins between activated and reverted HSCs

Clinical studies indicate that liver fibrosis could regress associated with decrease of cytokine and ECM production, increase of collagenase activity, and the disappearance of activated HSC after the etiological source (e.g., hepatitis B virus, hepatitis C virus, biliary obstruction, or alcohol) is removed^1^,^14^. Recent *in vitro*^15^,^16^,^17^,^18^ and *in vivo* studies^19^,^20^ provide new insights into the concept of reversibility of liver fibrosis, suggesting that the disappearance of activated HSCs is attributed not only to their apoptosis but also to reversion of their phenotype to a quiescent-like phenotype. Although some researchers have performed extensive microarray analysis of HSCs isolated at different time points after cessation of injury, whereas they did not identify a mediator or pathway that could actively return activated HSCs to a quiescent phenotype^20^. It was reported by She H *et al*.^6^ that MDI could convert activated HSCs to fat storage state even quiescent phenotype. Therefore, availability of MDI-induced model of changes in HSCs *in vitro* could help to understand the trans-differentiation process of HSCs reversion during liver fibrosis. The human hepatic cell line LX-2 was generated by XU L *et al*.^7^ and have extensively validated their similarities to human culture primary activated HSCs^8^. Such stable, immortal cell lines is an important tool to study mechanisms of hepatic fibrogenesis and test antifibrotic compounds. Hence, in the present study, we chose the adipocyte differentiation cocktail -MDI treating LX-2 to mimic quiescent HSC and identified some mediators and pathways that actively returns activated HSCs to a quiescent-like phenotype.

To our knowledge, this is the first comprehensive proteomic analysis of reverted HSCs that provided data for further study of HSCs. The present SILAC-based proteomic study identified a number of novel proteins associated with HSCs activation and reversion, extending our understanding of this reversion process. Based on GeneGO/MetaCore bioinformatics analysis, the most enriched biological function categories of up-regulated proteins in reverted HSCs were oxidation-reduction (74), organic acid metabolic (60), tricarboxylic acid cycle (17), carbohydrate metabolic (40), glucose metabolic (20), mitochondrial transport (14) and lipid oxidation process (10) (Fig. 2B). ALDH2 and NAMPT proteins were chosen for further validation by Western blotting (Fig. 3A) and demonstrated NAMPT protein could act as a possible marker for HCS reversion or quiescence. ALDH2 and NAMPT proteins belong to the oxidation-reduction biological function category. By microarray analysis, the transcript of ALDH2 were found to be down-regulated during the fibrogenesis process and potential used as novel serum markers of fibrosis^21^. NAMPT is a key nicotinamideadenime dinucleotide (NAD) biosynthetic enzyme in mammals, it has recently excited the scientific interest of researchers from diverse fields, including NAD biology, metabolic regulation and inflammation^22^. However, the top of enriched biological function categories of down-regulated proteins in reverted HSCs was cytoskeleton organization process (15) (Fig. 2B). Several proteins that are required for cytoskeleton organization were simultaneously down-regulated, including FLNA, EZRIN, VAMP8, and LASP1, *etc*. Moreover, muscle contraction (tropomyosin-1)^23^,^24^ and myoblast differentiation (Galectin-1)^25^ related proteins were also found to be down-regulated in reverted HSCs. Some of these novel proteins were further validated by Western blotting (Fig. 3A) and the results of these molecules closely correlate with the SILAC data. Conceivably, the regulation of cytoskeleton organization plays an important role in response to ECM remodeling and impact the development of the myofibroblast phenotype. From the data of the bioinformatics analysis, it can be found that activated HSCs reverted to quiescent-like HSCs, need to turn on the mechanism of oxidation reduction or metabolic process of organic acid, carbohydrate, etc. The process of reversion could affect the proteins related to the oxidation reduction, suggesting that MDI might induce a decline of oxidative stress^26^ and thus modifying the cytoskeleton^27^ and finally inhibiting the activation of HSCs.

Because our proteomic data were from the human hepatic stellate cell line not the human or animal primary hepatic stellate cells, some important results of bioinformatics analysis were further validated in animal model tissue and primary HSCs (Figs 3C,D,E and 4F). Our finding certified several cytoskeleton-associated proteins and oxidation- reduction related proteins as novel biomarkers of liver fibrosis, compared with the list of the largest scale proteomic profiling of rat activated HSCs^28^. Validated by Western blotting in CCl_4_-induced liver fibrosis development and regression, the expression of Filamin A and LASP1 protein were found increased as the expression of α- SMA and the expression of NAMPT in liver fibrosis was decreased (Fig. 3C). It indicated that Filamin A could serve as a possible marker for HCS activationact as α-SMA protein, further validated by immunohistochemistry in CCl_4_-induced fibrosis liver paraffin tissue section (Fig. 3D) and by immunofluorescence in cultured murine HSCs *in vitro* (Fig. 3E). Whereas, the interesting finding was the expression of CAV1 protein and 3 other altered proteins (STAT1, VAMP8, Mx1) in our list were diametrically opposite to the expression of which in the murine list^28^. Then three of these proteins (CAV1, STAT1 and VAMP8) were selected to validate and the results also closely in keeping with the SILAC data (Fig. 3A). Some reports illustrated CAV1 is a critical mediator of pulmonary fibrosis^29^,^30^,^31^. However, the role of CAV1 is little known about in HSCs even in liver fibrosis. In the present study, CAV1 may be act as an important role in reversion of activated HSCs.

### The roles of STAT1 in reversion of activated HSCs

The expression level of STAT1, as a member of the signal transducers and activators of transcription family, was suppressed during MDI-induced reverse stage of HSC in our proteomic study. In this sense, the status of STAT1 can be viewed as a key regulator for activated HSCs reversion, because it is a requirement to shut down for HSCs initiating the reversion program. Then in the following study, we provided evidence that STAT1 played two distinct roles in HSCs reversion. On the one hand, knockdown of STAT1 was involved in down-regulating the expression of cytoskeleton-associated proteins (Vimentin, LASP1 and VAMP8) and up-regulated the expression of oxidation- reduction associated proteins (ALDH2 and NAMPT) (Fig. 4D). On the other hand, knockdown of STAT1 could inhibit HSCs proliferation (Fig. 4B and C). HSCs normally undergo three distinct phases during injury induction: activation, proliferation and reversion. In the activation phase, the quienscent HSCs are activated in response to injury. Certain cytokines or chemokines released by inflammatory cells or other nearby cells could potentially server as the trigger. In the proliferation phase, the activated HSCs proliferate actively and generate a sufficient number of cells. In the reversion phase, activated HSCs differentiate and reverse to quiescent-like HSCs or apoptosis with resolution of injury. Consistently, our study shows that the expression of STAT1 is higher in acute hepatitis and liver fibrosis phase but lower in normal and recovery phase (Fig. 3C). Although STAT1 is rarely associated with proliferation, the activation of STAT1 even reduces HSCs proliferation^32^, a few of reports illustrated that STAT1 is required for myoblast or fibroblast proliferation^33^,^34^. Although it remains unclear what dictates these different outcomes, presumably, it may be in some particular cells that STAT1 could cooperate with other molecules/pathways to effects on cell proliferation. The involvement of STAT1 in HSCs proliferation is further supported by our following findings: Fludarabine, the specific inhibitor of STAT1, reduces activated HSCs proliferation and cytoskeleton-associated proteins (α-SMA and vimentin) (Fig. 4F). Furthermore, in our down-regulated proteomic list, ISG20 and Mx1 protein, related to IFN signaling pathway, could be activated by STAT1. Moreover, IFN-γ deficiency could inhibit the inflammatory response of Kupffer cells, subsequently suppress HSCs activation and liver fibrosis^35^. Collectively, these experiments highlighted the critical role of STAT1 in the progression of liver fibrosis and indicated that suppression of STAT1 promote the reversion of activated HSCs. Further investigation need to clarify the role of STAT1 or IFN signaling pathway in HSCs even more in liver fibrosis and recovery.

In brief, using SILAC technology to gain insight in the molecular changes provided the comprehensive proteome database of differentially expressed proteins between activated and reverted human HSCs. Bioinformatics analysis of proteome data added our understanding of the characteristics of reverted HSCs, such as oxidation-reduction, mitochondrial transport and lipid oxidation. Moreover, the present study cast new light on the role of STAT1 in reversion of activated HSCs and offered insight into the role of HSCs during liver injury. In conclusion, as the first comprehensive proteomic analysis of reverted HSCs, the data provided here will promote our understanding of the effect of HSC on liver fibrosis and recovery phase, and will accelerate the development of diagnostic and therapeutic strategies for liver fibrosis and other liver diseases.

## Material and Methods

### Cell lines and cultures

The human HSC cell line (LX-2)^7^ was kindly donated by Professor Scott L. Friedman (Mount Sinai School of Medicine, NY, USA) and Professor Lai Wei (Hepatology Institute, Peking University People’s Hospital, beijing, China). LX-2 cells were cultured in DMEM/F12 (Thermo Electron Corp., Australia) supplemented with 10% fetal bovine serum (Thermo Electron Corp., Australia), penicillin-streptomycin (Sigma, USA) at 37 °C in 5% CO_2_.

### Differential Expressed proteome Analysis Based on SILAC Labeling and LTQ-FT

SILAC labeling and LTQ-FT experiments (Thermo Electron, San Jose, CA, USA) were performed essentially as described^36^. LX-2 cells were divided into two groups to culture. Activated LX-2 cells were labeled with ^13^C_6_^15^N_2_-L-lysine (98% purity; Cambridge Isotope Laboratories, Andover, MA, USA); reverted LX-2 cells were labeled with ^12^C_6_^14^N_2_-L-lysine and induced to revert by adipocyte differentiation mixture (MDI) medium for 2 days^6^. Two parts of cells were harvested at the same time and lysed. Then equal amounts of proteins from two groups as described above was equally mixed (60 μg in total) and separated by 12% SDS-PAGE technology.

For the identification of proteins, the SDS-PAGE lanes were cut into 52 slices and digested as described previously by sun *et al*.^37^. Briefly, Gel pieces were reduced in 50 μl 10 mM DTT at 56 °C for 30 min, then the alkylation was performed by adding 50 μl 50 mM iodoacetamide (IAA), Next, 20 μl of the enzyme solution (0.01 μg/μl Trypsin, 25 mM NH_4_HCO_3_, 10% ACN) was added at 4 °C for 30 min. Finally, the supernatant was collected and dried.

The trypsin fragmented peptides were separated by Agilent 1100 Series binary HPLC system (Agilent Technologies, Palo Alto, CA) with RP C18 pre-column (GEAgel C18 SP-300-ODS-AP, particle size 5 μm, pore size 300 Å, 75 μm id × 10 cm long column, Jinouya, Beijing, China) and directly introduced to LTQ-FT mass spectrometer (Thermo Electron, San Jose, CA, USA). The mass spectrometer was operated as follow. In brief, each full MS scan (400–2000 m/z) was followed by ten data-dependent MS/MS scans on the most intense ions. The resolution was set as 100000 for survey scan.

In this study, FT data was analysed by Analyst MaxQuant (1.1.1.36) (Max Planck Institute of Biochemistry, Martinsried), the peptide mass tolerance was set as 10 ppm, FDR was less than 1%. More details are provided in the supporting information.

### Bioinformatics Analysis of Differentially Expressed Proteins

The bioinformatics analysis of the differentially expressed proteins was performed with GengGO/MetaCore software (https://portal.genego.com/). The lists of differential proteins were input into GengGO/MetaCore platform for identification of Subcellular localization and biological functional distribution. The false discovery rate was set to 0.05.

### Western Blotting

Western blotting analyses were performed on activated LX-2 and reverted LX-2 cells. 20 μg of total protein was separated on 12% SDS -PAGE gels. Proteins were transferred to nitrocellulose membranes (GE Healthcare, Germany). The membranes were blocked with 5% (w/v) nonfat milk in TBS tweenbuffer (TBST, 0.1%, v/v) for 1 h and then incubated with primary antibodies. A total of 13 commercial antibodies were used for Western blotting, including antibodies to a-SMA (1:200, Abcam, MA, USA), VIM (Vimentin, 1:1000, Abcam, MA, USA), STAT1 (Signal transducer and activator of transcription 1, 1:200, ProteinTech, Chicago, IL, USA), EZRIN (1:500, ProteinTech, Chicago, IL, USA), LGALS1 (Galectin-1, 1:1000, Abcam, MA, USA), LASP1 (LIM and SH3 domain protein 1, 1:1000, Abcam, MA, USA), VAMP8 (Vesicle-associated membrane protein 8, 1:1000, Epitomics Inc., Burlingame, CA, USA), FLNA (Filamin-A, 1:1000, Epitomics Inc., Burlingame, CA, USA), CAV1 (Caveolin-1, 1:1000, Abcam, MA, USA), NAMPT (Nicotinamide phosphoribosyltransferase, 1:200, Epitomics Inc., Burlingame, CA, USA), ALDH2 (Aldehyde dehydrogenase, 1:200, Abcam, MA, USA), GAPDH (1:5000, BioLegend, San Diego, CA) and β-actin (1:5000, BioLegend, San Diego, CA). GPADH and β-actin served as a loading control. After three subsequent washes in TBST at RT, the membranes were incubated with secondary HRP-antibodies (1:10,000, Jackson, West Grove, PA, USA) at RT for 1 h and washed again. Chemiluminescence detection was performed by ECL (Pierce, Rockford, USA).

### Mice Liver Fibrosis Model

Mice were housed at the Institutional Animal Care Facility of Beijing Proteome Research Center. All experiments were performed in accordance with relevant guidelines and regulations for laboratory animals. Eight week - old male C57BL6 mice (Beijing Laboratory Animal Center, Beijing, China) were used for Liver fibrosis model establishment. The animal use protocol was approved by the Animal Care Committee of Beijing Proteome Research Center. Liver fibrosis was induced by intraperitoneal administration of carbon tetrachloride (CCl_4_) in olive oil according to a previous study^13^. Histological liver analysis performed by an expert pathologist (I.F.) using a score from 0–3 for necrosis: 0. None, 1. Isolated hepatocytes, 2. Groups of hepatocytes, 3. Bridging. Ballooning of hepatocytes was scored as follows: 0. None, 1. Mild, 2. Moderate, 3. Severe. For each mouse, 10 fields at 40x magnification were analyzed and the average was calculated for each mouse.

### Immunohistochemistry and immunofluorescence

Mouse liver tissues were formalin fixed, embedded in paraffin and sectioned at 4 μm. Slides were routinely processed for immunohistochemically stained using the following antibodies: rabbit anti-αSMA (1:200, Abcam, MA, USA), rabbit anti- FLNA (1:1000, Abcam, MA, USA), mouse anti-VIM (1:500, Abcam, MA, USA). Visualization was perfomed using the Histostain-Plus Kit (ZSGB-BIO). For immunofluorescence studies, the isolated HSCs were cultured in DMEM/F12 supplemented with 10% fetal bovine serum for 1 month and kept stable proliferation rate and growth characteristics. These primary activated HSCs grown on cover slips were fixed and stained using the above antibodies. 20x pictures for each slip was analyzed using Image J software. The detail of mice liver fibrosis model and isolation of mice HSCs are provided in the supporting information.

### Small Interfering RNA Transfection, Cell Proliferation, Cell cycle Assays

The siRNA sequences specific for STAT1, as well as control siRNA were purchased from Genepharm. Transfection was performed according to the manufacturer’s recommendations using lipofactamine 2000 CD reagent (Invitrogen, Carlsbad, CA). After 48 h transfection, the efficiency of siRNA-mediated mRNA and protein degradation was assessed by real-time quantitative polymerase chain reaction (qPCR). The effects of STAT1 knockdown on HSC proliferation were measured by CCK-8 assay and Cell cycle assay followed by manufacturer’s instruction respectively.

### Fludarabine-mediated inhibition, Cell proliferation, Immunofluorescence

The LX-2 cells were treated with Fludarabine (50 μM, Sigma, USA) and incubated for 48 h. Proliferation of LX-2 cells treated with Fludarabine and MDI or without treated was detected by Cell Counting Kit-8 (Dojindo Molecular Technologies, Rockville, MD, USA) according to manufacturer’s instruction. For immunofluorescence studies, primary activated HSCs cultured for 1 month were grown on cover slips and treated with Fludarabine for 48 h or without, then incubated with the following antibodies: rabbit anti-αSMA (1:200, Abcam, MA, USA), mouse anti-Vimentin (1:500, Abcam, MA, USA). 20x pictures for each slip was analyzed using Image J software.

### Statistical Analysis

Data were expressed as the mean ± SD. Comparisons between groups were made by use of one-way ANOVA with SPSS 13.0 software. In GeneGO/MetaCore software analysis, the statistical data were generated by the software. Statistical significance was set at *p* < 0.05. Unless otherwise specified, all assays were performed in triplicate.

## Additional Information

**How to cite this article**: Zhang, H. *et al*. Quantitative Proteomic analysis on Activated Hepatic Stellate Cells reversion Reveal STAT1 as a key regulator between Liver Fibrosis and recovery. *Sci. Rep.* **7**, 44910; doi: 10.1038/srep44910 (2017).

**Publisher's note:** Springer Nature remains neutral with regard to jurisdictional claims in published maps and institutional affiliations.

## Supplementary Material

Supplementary Materials and Figures
