# Supplementary material for: Quantitative Proteomic analysis on Activated Hepatic Stellate Cells reversion Reveal STAT1 as a key regulator between Liver Fibrosis and recovery
Source: Sci Rep. 2017 Mar 21;7:44910. doi: 10.1038/srep44910 (PMC5359621; doi:10.1038/srep44910)

**Quantitative Proteomic analysis on Activated Hepatic Stellate Cells reversion Reveal** **STAT1** **as a key regulator between** **Liver Fibrosis and recovery**

**Author Names:**

Hongyu Zhang^1,2,3*^  [hongyu_zhang1@163.com](mailto:hongyu_zhang1@163.com)

Fangyan Chen^1*^  chenfangyan1986@163.com

Xu Fan^1^ fanxu2016@126.com

Cong Lin^1^  wuerlang1988@126.com

Yunwei hao^1^ [haoyw_bprc@163.com](mailto:haoyw_bprc@163.com)

Handong Wei^1^  [weihd@163.com](mailto:weihd@163.com)

Weiran Lin^1^ laop_530@163.com

Ying Jiang^1^ [jiangying304@hotmail.com](mailto:jiangying304@hotmail.com)

Fuchu He^1,4^ [hefc@nic.bmi.ac.cn](mailto:hefc@bmi.ac.cn)

^1^State Key Laboratory of Proteomics, Beijing Proteome Research Center, Beijing Institute of Radiation Medicine, Beijing, P. R. China, 100850.

^2^School of Life Sciences, Tsinghua University, Beijing, P. R. China, 100084.

^3^State Key Laboratory of Space Medicine Fundamentals and Application, China Astronaut Research and Training Center, Beijing, P. R. China, 100094.

^4^Institutes of Biomedical Sciences, Fudan University, Shanghai 200032, China

**Supplementary Materials and Methods**

***Reverted HSCs model***

LX-2 cells were seeded and grown until confluence, they were induced to differentiate by changing the medium to adipocyte differentiation mixture(MDI, 0.5 mM 3-isobutyl-1-methyxanthine (Sigma, USA), 1 µM dexamethasone (Sigma, USA), and 167 nM insulin (Sigma, USA) ) medium supplemented with or without 10% fetal bovine serum and incubated for 24 or 48 h as described previously[^1^](#_ENREF_1).

***Oil red O Staining***

Oil red O staining was performed to monitor degree of HSC differentiation as described previously[^1^](#_ENREF_1). Briefly, LX-2 cells were washed three times with PBS and then fixed by 4% paraformaldehyde for 5 minutes. The fixed LX-2 cells were incubated with Oil red O reagent (Sigma, USA) for one hour at room temperature and then washed with PBS. The stained fat droplets in the cells were visualized by light microscopy and photographed.

***Cell proliferation***

The cells were seeded in 96-well plates in DMEM/F12 supplemented with 10% fetal bovine serum, penicillin-streptomycin(1X). 24 hours later, the cells were treated with MDI and incubated for 48 h, and then cultured in serum-free medium for 18 (h). Proliferation of LX-2 cells treated with or without MDI was detected by Cell Counting Kit-8 (Dojindo Molecular Technologies, Rockville, MD, USA) according to manufacturer’s instruction.

***Cell cycle assay***

LX-2 cells were cultured in MDI with or without 10%FBS. At different time points, cells were collected, washed twice with 0.01 mol/L PBS, and fixed in 70% ethanol overnight at 4℃. The cells were subsequently washed once with PBS, resuspended in 200 µL Guava Cell Cycle Reagent (Millipore Company, Bedford, Mass) and incubated at room temperature for 30 minutes, shielded from light.  DNA histograms of the cell were assayed by flow cytometry (Millipore Company, Bedford, Mass) and analyzed. The experiments were repeated three times.

***SILAC Labeling***

LX-2 cells were divided into two groups to culture. one group of LX-2 cells were grown in L-lysine depleted DMEM/F12 medium (Thermo Electron Corp., Australia) supplemented with 100mg/L ^13^C_6_^15^N_2_-L-lysine (98% purity; Cambridge Isotope Laboratories, Andover, MA, USA) along with 10% Dialyzed Fetal Bovine Serum(Thermo Electron Corp., Australia)for 8 days until the complete incorporation of heavy lysine; another part of LX-2 cells were cultured for 6 days in DMEM/F12 medium with ^12^C_6_^14^N_2_-L-lysine and 10% Dialyzed Fetal Bovine Serum, then they were induced to differentiate by changing the medium to corresponding medium supplemented with 10% fetal bovine serum, 0.5 mM 3-isobutyl-1-methyxanthine (Sigma, USA), 1 µM dexamethasone (Sigma, USA), and 167 nM insulin (Sigma, USA) for 2 days. Two parts of cells were harvested at the same time after washed 3 times in ice cold PBS and stored at −80°C.

***Sample preparation***

The harvested cell pellets were resuspended in lysis buffer(4% SDS, 2% DTT, 120mM Tris-Cl (pH 6.8), Protease Inhibitor Cocktail) and lysed in homogenizer with 15sec and boiled with 3min, The lysates were then centrifuged at 20,000g for 30 minutes at 4°C after placed in room temperature with 30min. For proteomic studies, protein concentrations of lysates were determined in triplicate by RC DC protein quantification kit according to the manufacturer's instructions(based on the modified Lowry method, Bio-Rad Company ), then stored at -80 °C after dispensing for SDS-PAGE.

***SDS-PAGE separation***

Equal amounts of proteins from two groups of LX-2 cells labeled with ^13^C_6_^15^N_2_-L-lysine and LX-2 cells induced by MDI labeled with ^12^C_6_^14^N_2_-L-lysine as described above was equally mixed (60 μg in total) and separated by 12% SDS-PAGE technology. After electrophoresis, the gel was stained in Coomassie Brilliant Blue stain solution for 60min, and then decolored to clear protein bands with destaining solution (25% ethanol and 8% acetic acid aqueous solution).

***In -Gel digestion and peptide extraction***

For the identification of proteins, the whole SDS-PAGE lanes were cut into 52 slices and digested as described previously by sun *et al*. [^2^](#_ENREF_2) Briefly, the gel pieces were destained and rinsed with buffer containing 25 mM NH_4_HCO_3_ in 50% acetonitrile (ACN) for 20 min at room temperature (RT). This treatment was repeated until the blue faded. Gel pieces were shrunk in 100% v/v ACN for 5 min, and then discard the ACN, followed by the addition of 20 μl of the enzyme solution (0.01μg/μl Trypsin, 25mM NH_4_HCO_3_, 10% ACN) per gel piece at 4 °C for 30 min. When the enzyme solution was completely absorbed, add 15-20 μl 25mM NH_4_HCO_3_ solution to submerge the gel piece. Digestions were carried out overnight at 37 °C. the extracting solutionⅠ(5% TFA) and Ⅱ(2.5% TFA，50% ACN) was mix in eppendorf tubes and collected in sequence. The two extracting solutions were combined and dried in a vacuum centrifuge. The dried samples were dissolved in 20 μl 5% v/v ACN, and 0.1% v/v TFA for subsequent HPLC separation.

***LTQ-FT MS detection***

The trypsin fragmented peptides were analyzed by LTQ-FT mass sepectrometer (Thermo Electron, San Jose, CA, USA) equipped with Agilent 1100 Series binary HPLC system (Agilent Technologies, Palo Alto, CA) according to description of chen N *et al*[^3^](#_ENREF_3). The 20μL of peptide mixture in solvent A (2% v/v ACN, 98% v/v H_2_O, 0.1% v/v FA) was loaded onto the in-house RP C18 pre-column and desalted with solvent A. The separation was achieved by the 5-100% solvent B (80% v/v ACN, 20% v/v H_2_O, 0.1% v/v FA) for 90min with a flow rate of 300 nl/min. The eluent was introduced directly to LTQ-FT mass spectrometer.

***Data analysis***

Acquired raw files from mass spectrometer were analyzed by MaxQuant (v1.1.1.36)（Max Planck Institute of Biochemistry, Martinsried）with Mascot search engine, the parameters: heavy label lys 8; variable modifications were set as oxidation (Met) and acetylation (protein N terminus) ; carbamidomethyl (Cys) was chosen as fixed modification; Human International Protein Index (IPI) v3.78 was selected as database; enzyme was set on trypsin with no proline restriction, two missed cleavages; MS/MS tolerance was set to 0.5 Da; Peptide and protein false discovery rates (FDR) < 1%; maximum posterior error probability to 1; minimum of peptides and minimum of unique peptides to 2; minimum of peptide length to 6 amino acids. For quantification, default parameters were used.

***Isolation and Culture of mice HSCs***

Pathogen-free 12 week-old male C57/BL 6 mice (body weight,20-25g) used for HSC isolation were also cared for according to the Guide for the Care and Use of Laboratory Animals formulated by Institutional Animal Care and Use Committee Protocols. HSCs were isolated from mice livers by two-step digestion as described[^4^](#_ENREF_4). Density gradient separation yielded 0.5-1 million cells per animal. Trypan blue dye exclusion test showed that the isolated HSCs were more than 97% viable. The purity of HSCs was determined by autofluorescence of vitamin A. The isolated HSCs were cultured in DMEM/F12 supplemented with 10% fetal bovine serum, penicillin-streptomycin at 37 °C in 5% CO_2_.

**Supplemental Reference**

1 She, H., Xiong, S., Hazra, S. & Tsukamoto, H. Adipogenic transcriptional regulation of hepatic stellate cells. *The Journal of biological chemistry* **280**, 4959-4967, doi:10.1074/jbc.M410078200 (2005).

2 Sun, W. *et al.* Microwave-assisted protein preparation and enzymatic digestion in proteomics. *Molecular & cellular proteomics : MCP* **5**, 769-776, doi:10.1074/mcp.T500022-MCP200 (2006).

3 Chen, N. *et al.* Quantitative proteome analysis of HCC cell lines with different metastatic potentials by SILAC. *Proteomics* **8**, 5108-5118, doi:10.1002/pmic.200800280 (2008).

4 Liu, W. *et al.* Sample preparation method for isolation of single-cell types from mouse liver for proteomic studies. *Proteomics* **11**, 3556-3564, doi:10.1002/pmic.201100157 (2011).

**Supplemental Figure Legends**

Supplemental Figure.1 Confirmation of quiescent characteristics of MDI-induced LX-2 reversion model: (A) bright field, LX-2 cells connotes a morphology transition from proliferative, fibrogenic, and contractile myofibroblasts into lipid droplet-rich cells in MDI 1d and MDI+S 2d culture medium, (B) Oil Red O staining. cytoplasmic lipid droplets accumulation in MDI 1d and MDI+S 2d induced LX-2 cells(original magnification 200 ×). (C) cell proliferation. The mean OD value of cell proliferation was lower in MDI 1d (p<0.01) and MDI+S 2d LX-2 groups (p<0.01) than that in the control group respectively; Similarly，The G0/G1 phase  ratio  of LX-2 cells cultured in MDI 1d (P<0.01) and MDI+S 2d culture medium (P<0.05) was higher than that of control groups and the PI (proliferative  index, PI=S+G2/M) of LX-2 cells cultured in MDI 1d (P<0.01) and MDI+S 2d culture medium (P<0.05) was lower than that of control groups. (D) Western blotting showed that expression of α-SMA and Vimentin known  as  markers  of activated HSCs decreased in MDI 1d (p<0.01) and MDI+S 2d LX-2 groups (p<0.01) than that in the control group respectively. MDI 1d and MDI+S 2d LX-2 cells represent day1 and day2 activated HSCs. Each image is a representative of three independent experiments.

Supplemental Figure.2 Masson staining of liver sections. Liver fibrosis was induced by chronic intraperitoneal CCl_4_ administration (add one part olive oil to seven CCl_4_, 10μl CCl_4_/g body weight, twice a week). Mice were sacrificed at 4 weeks. The formalin/paraffin sections of liver tissue were stained with masson. The collagen deposition was stained blue. (A) Liver fibrosis stage 0-3 in the carbon tetrachloride (CCl_4_) mouse model, 1-4w. (B) Liver recovery stage in the carbon tetrachloride (CCl_4_) mouse model, 1-4w. Con: normal group, Oil: oil injected group. Each image is a representative of six animals in each group (original magnification 40×).

**Supplemental Table** 1. List of Up-regulated proteins in LX-2 cells treated with MDI for 48h, as determined by SILAC together with FT

| Majority Protein IDs | Razor Peptides | Unique Peptides | PEP | Protein Names | Gene Names | Ratio L/H Normalized |
| --- | --- | --- | --- | --- | --- | --- |
| IPI00465343 | 4 | 4 | 2.04E-08 | Alcohol dehydrogenase 1C | ADH1C | 35.696 |
| IPI00220045 | 2 | 2 | 1.79E-03 | DNA-directed RNA polymerase III 80 kDa polypeptide | KIAA1452 | 17.973 |
| IPI00443474 | 16 | 16 | 1.24E-83 | cDNA FLJ46716 fis, clone TRACH3018108, highly similar to 51 kDa FK506-binding protein | AIG6 | 12.045 |
| IPI00010130 | 5 | 5 | 0.00E+00 | Glutamate decarboxylase | GLNS | 8.662 |
| IPI00006079 | 1 | 1 | 3.31E-04 | Bcl-2-associated transcription factor 1 | BCLAF1 | 7.164 |
| IPI00015102 | 3 | 3 | 1.87E-05 | Activated leukocyte cell adhesion molecule | ALCAM | 6.887 |
| IPI00413500 | 1 | 1 | 1.32E-17 | COMM domain-containing protein 4 | COMMD4 | 5.777 |
| IPI00915357 | 7 | 7 | 4.55E-39 | cDNA FLJ60191, highly similar to Pyruvate dehydrogenase (lipoamide) kinase isozyme 3 (EC 2.7.11.2) | hCG_18655 | 4.198 |
| IPI00644712 | 3 | 3 | 2.21E-57 | 70 kDa subunit of Ku antigen | G22P1 | 4.012 |
| IPI00016801 | 20 | 20 | 1.96E-300 | Glutamate dehydrogenase 1, mitochondrial | GLUD | 3.474 |
| IPI00384938 | 2 | 2 | 1.97E-54 | Putative uncharacterized protein DKFZp686N02209 | DKFZp686N02209 | 3.321 |
| IPI00009346 | 1 | 1 | 6.25E-04 | Transmembrane protein 14C | C6orf53 | 3.296 |
| IPI00006663 | 13 | 13 | 0.00E+00 | Aldehyde dehydrogenase, mitochondrial | ALDH2 | 3.283 |
| IPI00009236 | 3 | 3 | 1.05E-55 | Caveolin-1 | CAV | 3.193 |
| IPI00012069 | 2 | 2 | 4.50E-07 | Azoreductase | DIA4 | 3.180 |
| IPI00152432 | 3 | 3 | 2.08E-10 | Alanine aminotransferase 2 | AAT2 | 3.107 |
| IPI00027166 | 3 | 3 | 2.74E-07 | CSC-21K | TIMP2 | 3.019 |
| IPI00008905 | 1 | 1 | 2.97E-04 | HLUG4 | UGT2B15 | 2.993 |
| IPI00376756 | 3 | 3 | 4.95E-06 | High mobility group protein 2a | HMG2A | 2.972 |
| IPI00293026 | 3 | 3 | 4.64E-22 | Elongation factor Tu GTP-binding domain-containing protein 1 | EFTUD1 | 2.905 |
| IPI00554521 | 8 | 8 | 9.25E-124 | Cell proliferation-inducing gene 15 protein | FTH | 2.867 |
| IPI00550239 | 6 | 6 | 8.93E-52 | Histone H1' | H1F0 | 2.866 |
| IPI00000684 | 8 | 7 | 1.03E-76 | AGX-1 | SPAG2 | 2.741 |
| IPI00024915 | 3 | 3 | 2.26E-08 | Alu corepressor 1 | ACR1 | 2.714 |
| IPI00027438 | 6 | 6 | 5.46E-89 | Flotillin-1 | FLOT1 | 2.695 |
| IPI00641579 | 1 | 1 | 4.57E-03 | cDNA FLJ37462 fis, clone BRAWH2011343, highly similar to COLD-INDUCIBLE RNA-BINDING PROTEIN | A18HNRNP | 2.669 |
| IPI00021924 | 2 | 2 | 1.31E-62 | Histone H1x | H1FX | 2.652 |
| IPI00747849 | 4 | 4 | 1.34E-16 | Sodium/potassium-dependent ATPase subunit beta-1 | ATP1B | 2.652 |
| IPI00909026 | 1 | 1 | 7.63E-09 | cDNA FLJ52689, highly similar to Homo sapiens mitochondrial ribosomal protein L21 (MRPL21), transcript variant 4, mRNA | MRPL21 | 2.609 |
| IPI00011107 | 17 | 16 | 2.12E-225 | ICD-M | IDH2 | 2.594 |
| IPI00022462 | 2 | 2 | 4.22E-45 | p90 | TFRC | 2.570 |
| IPI00789848 | 1 | 1 | 6.98E-04 | Putative uncharacterized protein IVD | IVD | 2.556 |
| IPI00045798 | 3 | 3 | 1.57E-23 | CL683 | GPX6 | 2.525 |
| IPI00847322 | 2 | 2 | 2.35E-05 | Superoxide dismutase | SOD2 | 2.475 |
| IPI00292695 | 2 | 2 | 1.21E-18 | Long-chain specific acyl-CoA dehydrogenase, mitochondrial | ACADL | 2.457 |
| IPI00215768 | 2 | 2 | 5.30E-07 | Gamma-ECS | GCLC | 2.450 |
| IPI00005260 | 2 | 2 | 2.80E-09 | Proteasome activator complex subunit 4 | KIAA0077 | 2.441 |
| IPI00032959 | 5 | 5 | 6.53E-45 | Glycerol-3-phosphate dehydrogenase 1-like protein | GPD1L | 2.413 |
| IPI00290279 | 4 | 4 | 1.39E-22 | Adenosine 5'-phosphotransferase | ADK | 2.410 |
| IPI00022887 | 4 | 4 | 6.72E-28 | Endoplasmic reticulum-Golgi intermediate compartment protein 1 | ERGIC1 | 2.389 |
| IPI00642432 | 3 | 2 | 3.06E-33 | Glutathione S-transferase M5 | GSTM5 | 2.376 |
| IPI00306719 | 2 | 2 | 7.59E-07 | cDNA FLJ55809 | CHID1 | 2.364 |
| IPI00027701 | 4 | 4 | 1.08E-62 | Butyryl-CoA dehydrogenase | ACADS | 2.349 |
| IPI00889196 | 2 | 2 | 3.59E-03 | Putative cytochrome b-c1 complex subunit Rieske-like protein 1 | UQCRFSL1 | 2.311 |
| IPI00024990 | 4 | 4 | 1.78E-65 | Aldehyde dehydrogenase family 6 member A1 | ALDH6A1 | 2.286 |
| IPI00401264 | 10 | 10 | 4.92E-77 | Endoplasmic reticulum resident protein 44 | ERP44 | 2.284 |
| IPI00003925 | 4 | 4 | 1.51E-223 | Pyruvate dehydrogenase E1 component subunit beta, mitochondrial | PDHB | 2.275 |
| IPI00003848 | 2 | 2 | 1.26E-14 | DnaJ homolog subfamily B member 4 | DNAJB4 | 2.268 |
| IPI00018873 | 5 | 5 | 2.11E-93 | Nicotinamide phosphoribosyltransferase | NAMPT | 2.257 |
| IPI00219729 | 9 | 9 | 5.82E-154 | Mitochondrial 2-oxoglutarate/malate carrier protein | SLC20A4 | 2.227 |
| IPI00025100 | 3 | 3 | 2.68E-41 | 2-oxoisovalerate dehydrogenase subunit alpha, mitochondrial | BCKDHA | 2.201 |
| IPI00220158 | 4 | 4 | 9.25E-17 | Alpha-adducin | ADD1 | 2.166 |
| IPI00011937 | 6 | 6 | 1.98E-186 | Antioxidant enzyme AOE372 | PRDX4 | 2.151 |
| IPI00293735 | 3 | 3 | 4.99E-06 | Elongator complex protein 1 | ELP1 | 2.142 |
| IPI00299088 | 2 | 2 | 8.30E-16 | Brain-specific angiogenesis inhibitor 1-associated protein 2 | BAIAP2 | 2.140 |
| IPI00872762 | 2 | 2 | 5.56E-04 | Succinyl-CoA ligase [GDP-forming] subunit alpha, mitochondrial | SUCLG1 | 2.136 |
| IPI00299402 | 16 | 16 | 1.71E-283 | Pyruvate carboxylase, mitochondrial | PC | 2.134 |
| IPI00783271 | 2 | 2 | 3.51E-06 | 130 kDa leucine-rich protein | LRP130 | 2.128 |
| IPI00945507 | 4 | 4 | 1.21E-16 | Putative uncharacterized protein SUCLG2 | SUCLG2 | 2.120 |
| IPI00176903 | 6 | 6 | 1.74E-261 | Cavin-1 | FKSG13 | 2.099 |
| IPI00030207 | 8 | 8 | 1.18E-128 | GDP-D-mannose dehydratase | GMDS | 2.088 |
| IPI00015911 | 4 | 4 | 2.68E-35 | Dihydrolipoamide dehydrogenase | DLD | 2.075 |
| IPI00790739 | 5 | 5 | 0.00E+00 | Aconitase 2, mitochondrial | ACO2 | 2.038 |
| IPI00382844 | 16 | 6 | 1.51E-245 | Aconitase | ACON | 2.037 |
| IPI00337495 | 18 | 18 | 2.76E-165 | Lysyl hydroxylase 2 | PLOD2 | 2.033 |
| IPI00556451 | 5 | 5 | 1.13E-16 | Electron transfer flavoprotein subunit beta | ETFB | 2.027 |
| IPI00022793 | 6 | 3 | 2.33E-23 | 3-ketoacyl-CoA thiolase | HADHB | 2.026 |
| IPI00012575 | 11 | 11 | 2.18E-165 | Pirin | PIR | 2.016 |
| IPI00879029 | 1 | 1 | 7.32E-05 | Unknown | Unknown | 2.001 |
| IPI00464979 | 2 | 2 | 3.76E-51 | ATP-specific succinyl-CoA synthetase subunit beta | SUCLA2 | 1.996 |
| IPI00154975 | 2 | 2 | 2.90E-07 | DnaJ homolog subfamily C member 9 | DNAJC9 | 1.985 |
| IPI00296259 | 3 | 3 | 3.38E-12 | Endoplasmic reticulum stress-response protein 25 | ERS25 | 1.958 |
| IPI00903226 | 14 | 11 | 6.14E-159 | cDNA FLJ46359 fis, clone TESTI4049786, highly similar to Hexokinase-1 (EC 2.7.1.1) | HK1 | 1.955 |
| IPI00923547 | 1 | 1 | 4.99E-06 | 60 kDa chaperonin | HSPD1 | 1.937 |
| IPI00018206 | 16 | 16 | 1.17E-187 | Aspartate aminotransferase, mitochondrial | GOT2 | 1.901 |
| IPI00746777 | 2 | 2 | 1.39E-04 | Alcohol dehydrogenase 5 | ADH5 | 1.895 |
| IPI00383581 | 15 | 15 | 4.65E-159 | Alpha-glucosidase 2 | G2AN | 1.879 |
| IPI00010154 | 10 | 10 | 0.00E+00 | Guanosine diphosphate dissociation inhibitor 1 | GDI1 | 1.871 |
| IPI00026328 | 5 | 5 | 2.72E-45 | Endoplasmic reticulum resident protein 18 | TLP19 | 1.870 |
| IPI00304417 | 5 | 5 | 2.90E-128 | Isocitrate dehydrogenase [NAD] subunit beta, mitochondrial | IDH3B | 1.865 |
| IPI00017726 | 3 | 3 | 2.87E-36 | 17-beta-hydroxysteroid dehydrogenase 10 | ERAB | 1.857 |
| IPI00018195 | 6 | 6 | 1.32E-56 | ERT2 | ERK1 | 1.856 |
| IPI00657648 | 1 | 1 | 1.56E-04 | Mammalian ependymin-related protein 1 | EPDR1 | 1.851 |
| IPI00168631 | 3 | 3 | 4.41E-45 | UPF0672 protein C3orf58 | C3orf58 | 1.851 |
| IPI00024934 | 3 | 3 | 2.43E-05 | Methylmalonyl-CoA isomerase | MUT | 1.849 |
| IPI00031558 | 3 | 3 | 5.70E-38 | Adenosine diphosphoribose pyrophosphatase | NUDT10 | 1.846 |
| IPI00305978 | 2 | 2 | 1.12E-53 | AFB1 aldehyde reductase 1 | AFAR | 1.840 |
| IPI00022334 | 7 | 7 | 4.87E-168 | Ornithine aminotransferase, hepatic form | OAT | 1.830 |
| IPI00020510 | 1 | 1 | 1.51E-62 | CDGSH iron sulfur domain-containing protein 1 | C10orf70 | 1.828 |
| IPI00550020 | 1 | 1 | 3.00E-25 | Parathymosin | PTMS | 1.826 |
| IPI00030877 | 2 | 2 | 4.67E-16 | 15 kDa selenoprotein | 15-Sep | 1.824 |
| IPI00926925 | 6 | 6 | 2.64E-31 | OGDH protein | OGDH | 1.819 |
| IPI00748807 | 1 | 1 | 1.27E-15 | 160 kDa nucleoporin | KIAA0197 | 1.817 |
| IPI00303476 | 20 | 20 | 0.00E+00 | ATP synthase subunit beta, mitochondrial | ATP5B | 1.812 |
| IPI00942266 | 4 | 4 | 8.75E-28 | SLC9A3R2 protein | SLC9A3R2 | 1.803 |
| IPI00908386 | 3 | 3 | 6.96E-10 | cDNA FLJ51127, highly similar to Alcohol dehydrogenase class 4 mu/sigma chain (EC 1.1.1.1) | ADH7 | 1.796 |
| IPI00017592 | 5 | 5 | 6.17E-43 | LETM1 and EF-hand domain-containing protein 1, mitochondrial | LETM1 | 1.792 |
| IPI00024662 | 1 | 1 | 2.85E-18 | Antigen p25 | CBX5 | 1.788 |
| IPI00031522 | 7 | 7 | 3.22E-28 | 78 kDa gastrin-binding protein | HADH | 1.779 |
| IPI00220487 | 2 | 2 | 1.43E-20 | ATP synthase subunit d, mitochondrial | ATP5H | 1.774 |
| IPI00294610 | 1 | 1 | 1.03E-06 | DnaJ homolog subfamily A member 3, mitochondrial | DNAJA3 | 1.772 |
| IPI00025366 | 7 | 7 | 3.02E-93 | Citrate synthase, mitochondrial | CS | 1.771 |
| IPI00149276 | 1 | 1 | 1.02E-03 | Brain and reproductive organ-expressed protein | BRCC45 | 1.764 |
| IPI00105407 | 2 | 2 | 2.10E-12 | Aldo-keto reductase family 1 member B10 | AKR1B10 | 1.761 |
| IPI00902560 | 8 | 8 | 7.35E-131 | Outer mitochondrial membrane protein porin 2 | VDAC2 | 1.753 |
| IPI00029557 | 5 | 5 | 1.42E-18 | GrpE protein homolog 1, mitochondrial | GREPEL1 | 1.752 |
| IPI00015842 | 2 | 2 | 8.09E-30 | Reticulocalbin-1 | RCN | 1.749 |
| IPI00303300 | 7 | 6 | 1.09E-17 | 65 kDa FK506-binding protein | FKBP10 | 1.740 |
| IPI00952680 | 4 | 4 | 2.97E-32 | Pitrilysin metalloproteinase 1 | KIAA1104 | 1.739 |
| IPI00217467 | 1 | 1 | 1.48E-224 | Histone H1.4 | H1F4 | 1.736 |
| IPI00376317 | 7 | 7 | 2.61E-148 | Autoantigen Ge-1 | EDC4 | 1.736 |
| IPI00023556 | 2 | 2 | 6.32E-05 | RNA polymerase II subunit A C-terminal domain phosphatase SSU72 | HSPC182 | 1.735 |
| IPI00060200 | 2 | 2 | 3.94E-24 | Aldose 1-epimerase | BLOCK25 | 1.733 |
| IPI00007611 | 2 | 2 | 1.76E-39 | ATP synthase subunit O, mitochondrial | ATP5O | 1.730 |
| IPI00027107 | 13 | 13 | 5.52E-172 | Elongation factor Tu, mitochondrial | TUFM | 1.729 |
| IPI00026215 | 5 | 5 | 7.26E-32 | DNase IV | FEN1 | 1.725 |
| IPI00011118 | 2 | 2 | 9.80E-15 | Putative uncharacterized protein RRM2 | RRM2 | 1.725 |
| IPI00290928 | 7 | 7 | 2.05E-86 | Guanine nucleotide-binding protein subunit alpha-13 | GNA13 | 1.720 |
| IPI00465315 | 3 | 3 | 5.57E-22 | Cytochrome c | CYC | 1.718 |
| IPI00396387 | 5 | 5 | 9.22E-27 | GTP-binding protein HSR1 | GNL1 | 1.711 |
| IPI00024913 | 2 | 2 | 1.06E-28 | ES1 protein homolog, mitochondrial | C21orf33 | 1.705 |
| IPI00884896 | 4 | 4 | 3.33E-60 | Gene associated with retinoic and interferon-induced mortality 12 protein | GRIM12 | 1.697 |
| IPI00296999 | 2 | 2 | 5.35E-18 | ATP synthase mitochondrial F1 complex assembly factor 2 | ATP12 | 1.695 |
| IPI00642238 | 6 | 6 | 8.30E-55 | Heterochromatin protein 1-binding protein 3 | HP1BP3 | 1.692 |
| IPI00152785 | 2 | 2 | 2.02E-190 | Histone H2B type 1-O | H2BFH | 1.691 |
| IPI00010810 | 6 | 6 | 5.19E-108 | Electron transfer flavoprotein subunit alpha, mitochondrial | ETFA | 1.690 |
| IPI00030363 | 2 | 2 | 3.09E-03 | Acetoacetyl-CoA thiolase | ACAT | 1.683 |
| IPI00220150 | 7 | 7 | 5.13E-40 | Isocitrate dehydrogenase [NAD] subunit gamma, mitochondrial | IDH3G | 1.681 |
| IPI00017367 | 9 | 9 | 1.58E-276 | Radixin isoform b | hCG_39182 | 1.681 |
| IPI00010080 | 3 | 3 | 1.82E-15 | Oxidative stress-responsive 1 protein | KIAA1101 | 1.680 |
| IPI00295940 | 3 | 3 | 1.06E-17 | cDNA FLJ55508, highly similar to Sad1/unc-84-like protein 2 | UNC84B | 1.680 |
| IPI00472939 | 3 | 3 | 7.27E-18 | Microsomal signal peptidase 25 kDa subunit | KIAA0102 | 1.674 |
| IPI00031131 | 2 | 2 | 5.09E-23 | Adipocyte plasma membrane-associated protein | APMAP | 1.673 |
| IPI00924713 | 4 | 4 | 1.65E-23 | cDNA, FLJ93994, highly similar to Homo sapiens phosphate cytidylyltransferase 1, choline, alpha isoform (PCYT1A), mRNA | PCYT1A | 1.664 |
| IPI00440493 | 20 | 20 | 0.00E+00 | ATP synthase subunit alpha, mitochondrial | ATP5A | 1.662 |
| IPI00386271 | 10 | 6 | 1.67E-128 | Calcium-binding mitochondrial carrier protein Aralar1 | ARALAR1 | 1.658 |
| IPI00000030 | 3 | 3 | 3.74E-08 | PP2A B subunit isoform B56-delta | PPP2R5D | 1.656 |
| IPI00022824 | 7 | 7 | 3.99E-74 | Bax-interacting factor 1 | CGI-61 | 1.653 |
| IPI00177728 | 4 | 4 | 3.87E-71 | CNDP dipeptidase 2 | CN2 | 1.652 |
| IPI00027175 | 2 | 2 | 3.39E-22 | 22 kDa protein | SRI | 1.649 |
| IPI00020539 | 2 | 2 | 3.76E-50 | 5-oxoprolyl-peptidase | PGPEP1 | 1.647 |
| IPI00073779 | 2 | 2 | 1.73E-11 | 28S ribosomal protein S28, mitochondrial | HDCMD11P | 1.646 |
| IPI00219525 | 6 | 6 | 1.50E-149 | 6-phosphogluconate dehydrogenase, decarboxylating | PGD | 1.646 |
| IPI00384280 | 3 | 3 | 6.92E-37 | Prenylcysteine lyase | KIAA0908 | 1.645 |
| IPI00024919 | 5 | 5 | 4.39E-30 | Antioxidant protein 1 | AOP1 | 1.644 |
| IPI00021338 | 7 | 7 | 4.85E-29 | 70 kDa mitochondrial autoantigen of primary biliary cirrhosis | DLAT | 1.632 |
| IPI00028122 | 4 | 4 | 1.56E-33 | CLL-associated antigen KW-7 | DFS70 | 1.632 |
| IPI00291006 | 12 | 12 | 0.00E+00 | Malate dehydrogenase, mitochondrial | MDH2 | 1.626 |
| IPI00023064 | 1 | 1 | 4.68E-05 | Hormone-regulated proliferation-associated protein of 20 kDa | C6orf66 | 1.622 |
| IPI00030357 | 4 | 4 | 1.70E-06 | Dihydrofolate reductase | DHFR | 1.617 |
| IPI00027230 | 35 | 28 | 0.00E+00 | 94 kDa glucose-regulated protein | GRP94 | 1.611 |
| IPI00008485 | 4 | 4 | 4.06E-47 | Citrate hydro-lyase | ACO1 | 1.607 |
| IPI00641924 | 1 | 1 | 1.21E-04 | 28S ribosomal protein S9, mitochondrial | MRPS9 | 1.605 |
| IPI00008454 | 9 | 9 | 2.40E-80 | APOBEC1-binding protein 2 | DNAJB11 | 1.604 |
| IPI00394767 | 1 | 1 | 3.83E-03 | Carbonate dehydratase XIII | CA13 | 1.604 |
| IPI00005614 | 21 | 20 | 0.00E+00 | Beta-II spectrin | SPTB2 | 1.604 |
| IPI00007691 | 2 | 2 | 3.48E-03 | Hematopoietic stem/progenitor cell protein 172 | CGI-104 | 1.602 |
| IPI00306516 | 3 | 3 | 1.88E-35 | Mitochondrial import inner membrane translocase subunit TIM44 | MIMT44 | 1.601 |
| IPI00604759 | 1 | 1 | 3.16E-09 | Fumarylacetoacetate hydrolase domain containing 1 | AC012180.10-003 | 1.601 |
| IPI00029046 | 4 | 4 | 1.07E-21 | Malectin | KIAA0152 | 1.600 |
| IPI00000821 | 2 | 2 | 7.84E-07 | 39S ribosomal protein L16, mitochondrial | MRPL16 | 1.600 |
| IPI00004358 | 15 | 8 | 1.35E-132 | Glycogen phosphorylase, brain form | PYGB | 1.599 |
| IPI00470610 | 2 | 2 | 4.62E-81 | Pyrroline-5-carboxylate reductase 2 | PYCR2 | 1.599 |
| IPI00219217 | 6 | 6 | 3.90E-89 | LDH heart subunit | LDHB | 1.598 |
| IPI00465179 | 8 | 6 | 3.33E-71 | cDNA FLJ44241 fis, clone THYMU3008436, highly similar to 6-phosphofructokinase, muscle type (EC 2.7.1.11) | PFKM | 1.597 |
| IPI00003326 | 2 | 2 | 3.42E-32 | ADP-ribosylation factor-like protein 2 | ARL2 | 1.592 |
| IPI00216308 | 16 | 16 | 0.00E+00 | Outer mitochondrial membrane protein porin 1 | VDAC | 1.591 |
| IPI00397860 | 2 | 2 | 2.06E-14 | Cytochrome b5 | CYB5 | 1.591 |
| IPI00021692 | 3 | 1 | 3.92E-10 | Muscleblind-like protein 1 | EXP | 1.591 |
| IPI00745792 | 1 | 1 | 8.74E-05 | NmrA-like family domain-containing protein 1 | HSCARG | 1.580 |
| IPI00550364 | 3 | 3 | 1.37E-11 | Glucose phosphomutase 2 | MSTP006 | 1.580 |
| IPI00646762 | 2 | 2 | 2.01E-05 | Putative uncharacterized protein NUDT5 | NUDT5 | 1.579 |
| IPI00147874 | 7 | 7 | 2.11E-62 | N-acetylneuraminate synthase | NANS | 1.578 |
| IPI00514926 | 1 | 1 | 4.46E-03 | Guanylate kinase 1 | GUK1 | 1.576 |
| IPI00828189 | 4 | 4 | 5.69E-68 | L-isoaspartyl protein carboxyl methyltransferase | PCMT1 | 1.574 |
| IPI00217277 | 1 | 1 | 6.75E-115 | Mitochondrial dicarboxylate carrier | DIC | 1.573 |
| IPI00061245 | 1 | 1 | 3.92E-09 | 28S ribosomal protein S10, mitochondrial | MRPS10 | 1.571 |
| IPI00016608 | 2 | 2 | 3.62E-07 | Membrane protein p24A | RNP24 | 1.567 |
| IPI00291764 | 2 | 2 | 1.04E-234 | Histone H2A type 1 | H2AFC | 1.565 |
| IPI00418313 | 6 | 6 | 5.03E-30 | Double-stranded RNA-binding protein 76 | DRBF | 1.564 |
| IPI00012828 | 2 | 2 | 9.47E-10 | 3-ketoacyl-CoA thiolase, peroxisomal | ACAA | 1.563 |
| IPI00001541 | 1 | 1 | 3.96E-05 | Mitochondrial import inner membrane translocase subunit Tim9 | TIM9 | 1.554 |
| IPI00003482 | 2 | 2 | 2.77E-07 | 2,4-dienoyl-CoA reductase [NADPH] | DECR | 1.552 |
| IPI00655631 | 3 | 3 | 5.62E-09 | DNA polymerase | POLD | 1.550 |
| IPI00553067 | 4 | 4 | 1.87E-07 | Coiled-coil domain-containing protein 132 | CCDC132 | 1.543 |
| IPI00879810 | 24 | 24 | 4.42E-254 | Putative uncharacterized protein SPTAN1 | SPTAN1 | 1.542 |
| IPI00420014 | 11 | 11 | 1.51E-95 | Activating signal cointegrator 1 complex subunit 3-like 1 | ASCC3L1 | 1.540 |
| IPI00297572 | 10 | 10 | 7.16E-108 | Intron-binding protein aquarius | AQR | 1.540 |
| IPI00640276 | 1 | 1 | 1.18E-28 | EF-hand domain-containing family member A1 | EFHA1 | 1.540 |
| IPI00004839 | 5 | 5 | 1.77E-37 | Crk-like protein | CRKL | 1.539 |
| IPI00217465 | 14 | 1 | 2.88E-240 | Histone H1.2 | H1F2 | 1.536 |
| IPI00013475 | 4 | 4 | 0.00E+00 | Tubulin beta-2A chain | TUBB2 | 1.535 |
| IPI00296053 | 6 | 6 | 1.87E-64 | Fumarate hydratase, mitochondrial | FH | 1.535 |
| IPI00030275 | 13 | 13 | 3.72E-158 | Heat shock protein 75 kDa, mitochondrial | HSP75 | 1.533 |
| IPI00026154 | 3 | 3 | 1.23E-13 | cDNA FLJ59211, highly similar to Glucosidase 2 subunit beta | G19P1 | 1.529 |
| IPI00166955 | 3 | 3 | 1.49E-20 | Protein canopy homolog 4 | CNPY4 | 1.528 |
| IPI00014376 | 1 | 1 | 8.72E-15 | Ras-related protein Rab-22B | RAB22B | 1.527 |
| IPI00103525 | 2 | 2 | 4.57E-03 | Paraspeckle component 1 | PSP1 | 1.527 |
| IPI00784154 | 26 | 25 | 0.00E+00 | 60 kDa chaperonin | HSP60 | 1.523 |
| IPI00329373 | 1 | 1 | 2.87E-07 | Protein P117 | C19orf70 | 1.522 |
| IPI00930710 | 5 | 5 | 1.11E-123 | Mitochondrial aspartate-glutamate carrier protein | hCG_40633 | 1.521 |
| IPI00374657 | 4 | 3 | 5.76E-33 | 33 kDa VAMP-associated protein | VAP33 | 1.520 |
| IPI00217468 | 6 | 6 | 1.17E-108 | Histone H1.5 | H1F5 | 1.518 |
| IPI00219575 | 1 | 1 | 5.72E-11 | Bleomycin hydrolase | BLMH | 1.516 |
| IPI00014843 | 3 | 3 | 7.28E-04 | CARMIL homolog | CARMIL | 1.510 |
| IPI00921996 | 10 | 7 | 2.48E-52 | Stathmin | LAP18 | 1.509 |
| IPI00555597 | 1 | 1 | 4.93E-05 | cDNA FLJ32471 fis, clone SKNMC2000322, highly similar to Peptidyl-tRNA hydrolase 2, mitochondrial (EC 3.1.1.29) | BIT1 | 1.507 |
| IPI00304171 | 11 | 11 | 4.61E-173 | Core histone macro-H2A.1 | H2AFY | 1.504 |
| IPI00743772 | 2 | 2 | 7.22E-04 | COMM domain-containing protein 7 | C20orf92 | 1.503 |
| IPI00006725 | 6 | 6 | 4.44E-17 | 100 kDa U5 snRNP-specific protein | DDX23 | 1.501 |
| IPI00013167 | 2 | 2 | 5.47E-29 | 28S ribosomal protein S25, mitochondrial | MRPS25 | 1.501 |
| IPI00031655 | 6 | 6 | 8.30E-33 | Dermal papilla-derived protein 9 | DERP9 | 1.501 |

This table contains the 212 proteins that displaying more than 1.5-fold up-regulation in reverted human hepatic stellate cells in SILAC experiments. The International Protein Index (IPI) accession number, Razor peptides, Unique Peptides, PEP, protein name, gene name and Ratio L/H Normalized of each protein are provided here. The proteins are listed in a descending order according to their fold change. PEP: Posterior error probability, Razor peptides: Razor peptides are non-unique peptides assigned to the protein group with the most other peptides (Occam's razor principle).

**Supplemental** Table 2. List of Down-regulated proteins in LX-2 cells treated with MDI for 48h, as determined by SILAC together with FT

| Majority Protein IDs | Razor Peptides | Unique Peptides | PEP | Protein Names | Gene Names | Ratio L/H Normalized |
| --- | --- | --- | --- | --- | --- | --- |
| IPI00026833 | 6 | 6 | 3.15E-60 | Adenylosuccinate synthetase isozyme 2 | ADSS | 0.665 |
| IPI00018522 | 8 | 8 | 1.17E-42 | Interferon receptor 1-bound protein 4 | HMT2 | 0.665 |
| IPI00032140 | 14 | 14 | 1.58E-279 | 47 kDa heat shock protein | CBP1 | 0.663 |
| IPI00549248 | 8 | 8 | 2.12E-235 | Nucleolar phosphoprotein B23 | NPM | 0.659 |
| IPI00328154 | 5 | 5 | 1.61E-42 | NEDD8-activating enzyme E1 catalytic subunit | UBA3 | 0.657 |
| IPI00855846 | 1 | 1 | 7.03E-26 | UPF0727 protein C6orf115 | C6orf115 | 0.653 |
| IPI00025491 | 22 | 11 | 0.00E+00 | ATP-dependent RNA helicase eIF4A-1 | DDX2A | 0.653 |
| IPI00003527 | 3 | 3 | 1.12E-13 | Ezrin-radixin-moesin-binding phosphoprotein 50 | NHERF | 0.653 |
| IPI00296635 | 2 | 2 | 4.80E-11 | 1,4-alpha-glucan-branching enzyme | GBE1 | 0.649 |
| IPI00021570 | 1 | 1 | 1.33E-12 | Endothelial differentiation-related factor 1 | EDF1 | 0.648 |
| IPI00414860 | 4 | 4 | 4.56E-60 | 60S ribosomal protein L37a | RPL37A | 0.647 |
| IPI00011268 | 2 | 2 | 2.08E-07 | Autoantigen p542 | HNRPCL2 | 0.644 |
| IPI00008524 | 23 | 14 | 0.00E+00 | Polyadenylate-binding protein 1 | PAB1 | 0.637 |
| IPI00012535 | 11 | 11 | 5.32E-66 | DnaJ homolog subfamily A member 1 | DNAJ2 | 0.635 |
| IPI00386803 | 6 | 6 | 8.36E-51 | LIM and SH3 domain protein 1 | LASP1 | 0.631 |
| IPI00329441 | 1 | 1 | 1.08E-06 | Ras-related protein Rab-41 | RAB41 | 0.630 |
| IPI00102864 | 6 | 6 | 1.69E-33 | Hexokinase type II | HK2 | 0.629 |
| IPI00290142 | 9 | 9 | 1.71E-83 | CTP synthase 1 | CTPS | 0.629 |
| IPI00956559 | 4 | 4 | 9.52E-29 | EIF4G1 protein | EIF4G1 | 0.621 |
| IPI00550746 | 5 | 5 | 1.83E-11 | Nuclear distribution protein C homolog | NUDC | 0.619 |
| IPI00022228 | 24 | 24 | 4.99E-218 | High density lipoprotein-binding protein | HBP | 0.617 |
| IPI00010414 | 3 | 3 | 2.28E-35 | C-terminal LIM domain protein 1 | CLIM1 | 0.615 |
| IPI00797126 | 4 | 3 | 1.95E-238 | Alpha-NAC | HSD48 | 0.606 |
| IPI00009322 | 14 | 14 | 3.90E-74 | Cell proliferation-inducing gene 53 protein | KIAA0093 | 0.605 |
| IPI00030911 | 3 | 3 | 9.07E-11 | Endobrevin | VAMP8 | 0.600 |
| IPI00843975 | 11 | 11 | 7.84E-221 | Cytovillin | EZR | 0.599 |
| IPI00015953 | 8 | 8 | 5.95E-86 | DEAD box protein 21 | DDX21 | 0.599 |
| IPI00012479 | 1 | 1 | 1.61E-31 | Alpha-NAC pseudogene 1 | FKSG17 | 0.598 |
| IPI00883896 | 5 | 5 | 1.83E-22 | Epithelial protein lost in neoplasm | EPLIN | 0.587 |
| IPI00514587 | 10 | 10 | 8.38E-170 | Seryl-tRNA synthetase | RP11-352P4.2-004 | 0.586 |
| IPI00867509 | 5 | 5 | 7.87E-61 | Coronin-1C_i2 protein | CORO1C | 0.585 |
| IPI00221035 | 6 | 6 | 1.17E-119 | RNA polymerase B transcription factor 3 | BTF3 | 0.582 |
| IPI00030781 | 10 | 1 | 2.27E-102 | Signal transducer and activator of transcription 1-alpha/beta | STAT1 | 0.581 |
| IPI00015077 | 2 | 2 | 1.05E-55 | A121 | EIF1 | 0.571 |
| IPI00017184 | 21 | 18 | 1.16E-283 | EH domain-containing protein 1 | CDABP0131 | 0.568 |
| IPI00329719 | 8 | 8 | 4.57E-95 | Myosin-Id | KIAA0727 | 0.563 |
| IPI00002895 | 2 | 2 | 2.63E-04 | DRB sensitivity-inducing factor 14 kDa subunit | SPT4H | 0.561 |
| IPI00026689 | 9 | 7 | 1.70E-60 | Cell division control protein 2 homolog | CDC2 | 0.552 |
| IPI00216654 | 4 | 4 | 1.71E-13 | 140 kDa nucleolar phosphoprotein | KIAA0035 | 0.541 |
| IPI00068506 | 4 | 4 | 2.22E-26 | ATP-binding cassette, sub-family F (GCN20), member 2, isoform CRA_d | ABCF2 | 0.536 |
| IPI00000811 | 2 | 2 | 1.42E-12 | Macropain delta chain | LMPY | 0.532 |
| IPI00219005 | 8 | 8 | 2.16E-58 | 51 kDa FK506-binding protein | FKBP4 | 0.529 |
| IPI00554777 | 9 | 9 | 3.18E-32 | Asparagine synthetase [glutamine-hydrolyzing] | ASNS | 0.524 |
| IPI00333541 | 34 | 31 | 3.25E-276 | Actin-binding protein 280 | FLN | 0.518 |
| IPI00219219 | 4 | 4 | 8.42E-125 | 14 kDa laminin-binding protein | LGALS1 | 0.514 |
| IPI00015952 | 7 | 7 | 1.63E-27 | cDNA FLJ59571, highly similar to Eukaryotic translation initiation factor 4gamma 2 | DAP5 | 0.512 |
| IPI00783872 | 3 | 3 | 3.11E-43 | Caprin-1 | CAPRIN1 | 0.497 |
| IPI00028296 | 3 | 3 | 4.48E-14 | Calcium/calmodulin-dependent protein kinase type 1 | CAMK1 | 0.478 |
| IPI00020965 | 3 | 3 | 1.51E-39 | UbcH2 | UBE2H | 0.465 |
| IPI00015891 | 1 | 1 | 1.23E-05 | Prefoldin subunit 4 | PFD4 | 0.428 |
| IPI00795490 | 1 | 1 | 6.41E-12 | cDNA FLJ56490, highly similar to Serpin I2 | MEPI | 0.426 |
| IPI00013002 | 4 | 4 | 9.68E-13 | UbcH10 | UBCH10 | 0.408 |
| IPI00014377 | 2 | 2 | 8.97E-09 | Ras-related protein Rab-32 | RAB32 | 0.370 |
| IPI00100748 | 4 | 4 | 2.89E-42 | Heat shock protein-binding protein 1 | HSPBP | 0.352 |
| IPI00291463 | 4 | 4 | 3.94E-27 | Cytomegalovirus-induced gene 5 protein | CIG5 | 0.315 |
| IPI00215920 | 4 | 4 | 1.26E-06 | ADP-ribosylation factor 6 | ARF6 | 0.305 |
| IPI00167949 | 5 | 5 | 6.36E-97 | Interferon-induced GTP-binding protein Mx1 | MX1 | 0.279 |
| IPI00941607 | 1 | 1 | 9.27E-04 | Negative elongation factor E | NELFE | 0.256 |
| IPI00027799 | 1 | 1 | 2.04E-04 | Protein FAM107B | C10orf45 | 0.223 |
| IPI00216135 | 5 | 2 | 4.21E-151 | Alpha-tropomyosin | C15orf13 | 0.176 |
| IPI00413368 | 2 | 2 | 4.20E-06 | Protein FAM25 | FAM25A | 0.169 |
| IPI00647246 | 2 | 2 | 1.63E-04 | Estrogen-regulated transcript 45 protein | HEM45 | 0.163 |

This table contains the 61 proteins that displaying more than 1.5-fold down-regulation in reverted human hepatic stellate cells in SILAC experiments. The International Protein Index (IPI) accession number, Razor peptides, Unique Peptides, PEP, protein name, gene name and Ratio L/H Normalized of each protein are provided here. The proteins are listed in a descending order according to their fold change. PEP: Posterior error probability, Razor peptides: Razor peptides are non-unique peptides assigned to the protein group with the most other peptides (Occam's razor principle).


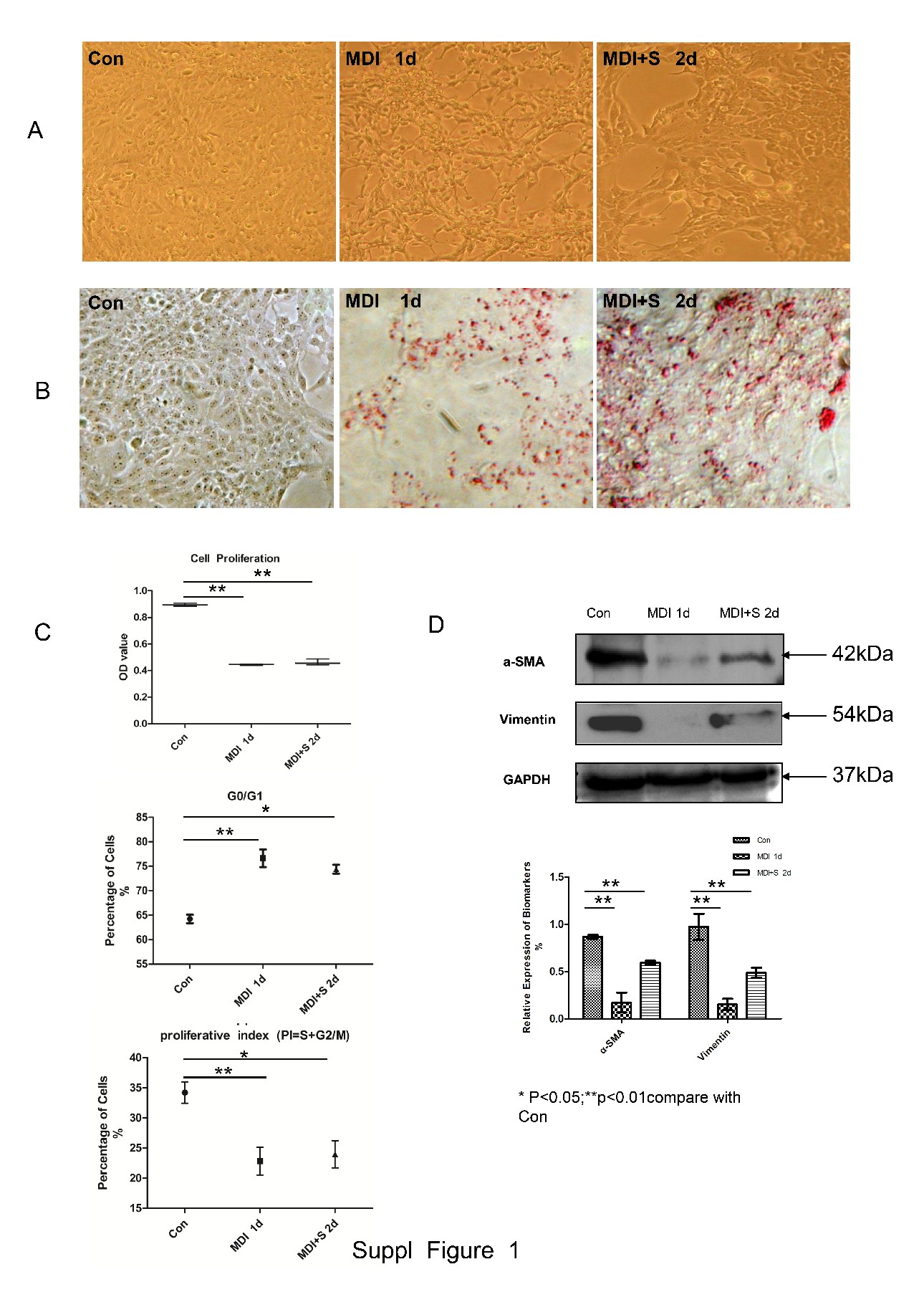


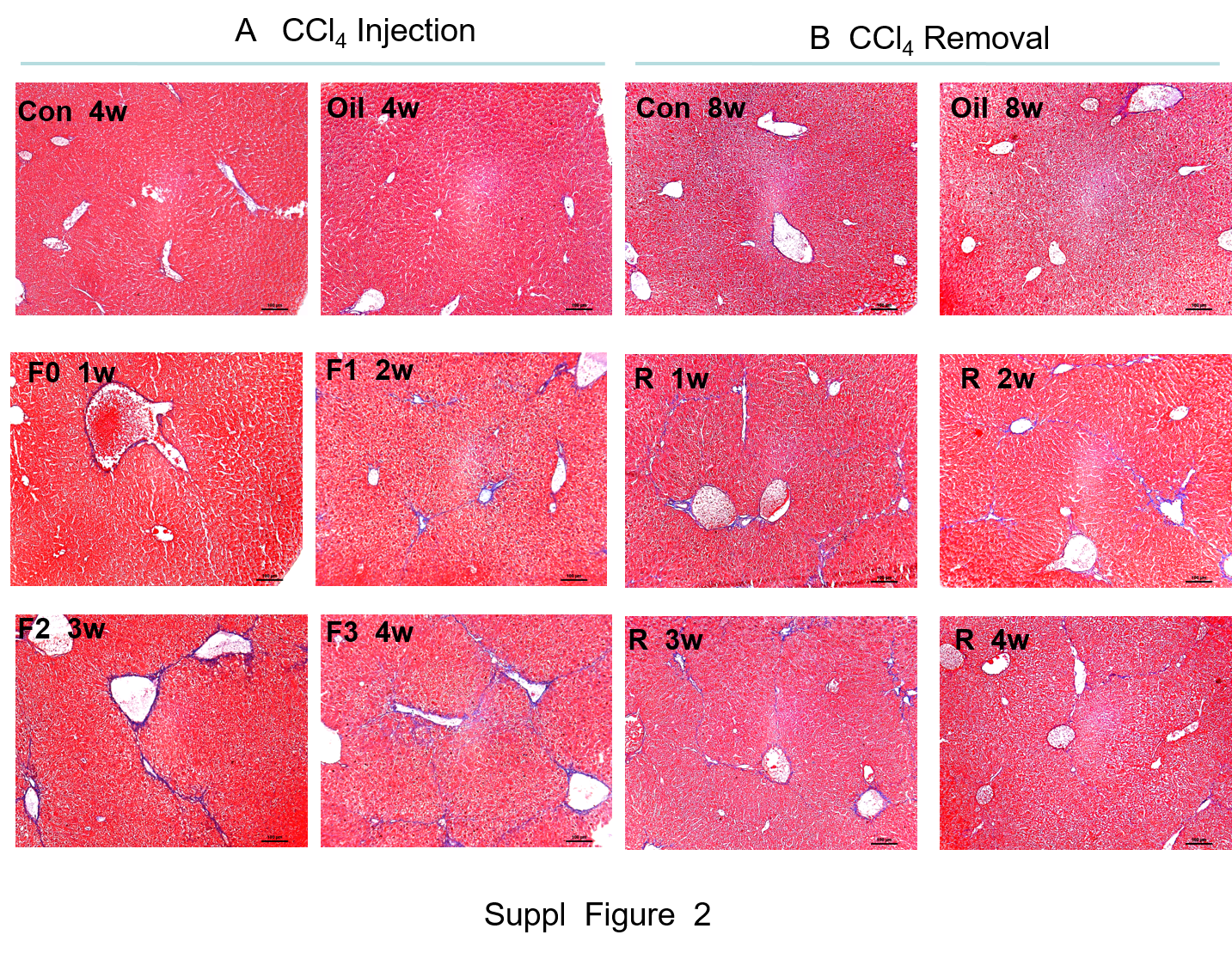

Supplement: Supplementary Materials and Figures [file srep44910-s1.doc]
